# Activating the PGC-1*α*/TERT Pathway by Catalpol Ameliorates Atherosclerosis via Modulating ROS Production, DNA Damage, and Telomere Function: Implications on Mitochondria and Telomere Link

**DOI:** 10.1155/2018/2876350

**Published:** 2018-06-25

**Authors:** Yukun Zhang, Changyuan Wang, Yue Jin, Qining Yang, Qiang Meng, Qi Liu, Yongguo Dai, Lifei Cai, Zhihao Liu, Kexin Liu, Huijun Sun

**Affiliations:** Department of Clinical Pharmacology, College of Pharmacy, Dalian Medical University, Dalian, China

## Abstract

Catalpol, an iridoid glucoside, has been found present in large quantities in the root of *Rehmannia glutinosa* L. and showed a strong antioxidant capacity in the previous study. In the present work, the protective effect of catalpol against AS via inhibiting oxidative stress, DNA damage, and telomere shortening was found in LDLr^−/−^ mice. This study also shows that activation of the peroxisome proliferator-activated receptor-*γ* coactivator-1*α* (PGC-1*α*)/telomerase reverse transcriptase (TERT) pathway, which is the new link between mitochondria and telomere, was involved in the protective effects of catalpol. Further, by using PGC-1*α* or TERT siRNA in oxLDL-treated macrophages, it is proved that catalpol reduced oxidative stress, telomere function, and related DNA damage at least partly through activating the PGC-1*α*/TERT pathway. Moreover, dual luciferase activity assay-validated catalpol directly enhanced PGC-1*α* promoter activity. In conclusion, our study revealed that the PGC-1*α*/TERT pathway might be a possible therapeutic target in AS and catalpol has highly favorable characteristics for the treatment of AS via modulating this pathway.

## 1. Introduction

Atherosclerotic cardiovascular disease is a kind of aging-related degenerative disease comprising of various disorders of the heart and blood vessels [1]. A well-documented hypothesis indicated that atherosclerosis (AS) is closely related to oxidative stress and aging. Moreover, oxidative stress and aging were considered to be the major causes of inflammation, DNA damage, and telomere dysfunction and shortening [2–5]. Macrophage plays a pivotal role in swallowing the plaque accumulation of lipids and apoptotic cells, maintaining the dynamic balance within the plaque of AS. However, effective therapeutic interventions against AS provided by pharmacotherapy via modulating macrophage ROS accumulation, DNA damage, and telomere function are still currently limited.

Peroxisome proliferator-activated receptor-*γ* coactivator *α* (PGC-1*α*) is an auxotrophic transcriptional factor capable of interacting with a transcription factor or another coactivator to increase the transcription efficiency of the target gene. PGC-1*α* modulates diverse biological processes, ranging from metabolism, oxidative stress resistance, inflammation, aging, and redox balance to apoptosis [6–8]. Likewise, PGC-1*α* expression is profoundly repressed in AS, thereby increasing ROS accumulation and cell apoptosis [9–11]. Although the repression of PGC-1*α* expression during AS was found, its specific roles as well as the detailed mechanisms are largely unknown.

Atherosclerotic cardiovascular disease also emerges coincidentally from cell senescence and organismal aging [5]. Telomere length is closely associated with cell senescence [12–14]. Telomerase reverse transcriptase (TERT), the protein subunit of telomerase, maintains telomere ends during DNA replication by catalyzing the addition of short telomere repeats [15]. A recent report from Mark Zurek demonstrates that TERT is active in cells of the cardiovascular system including cardiac myocytes, endothelial cells, smooth muscle cells, and fibroblasts, and regulation on TERT may be a potential target greatly contributing to the prevention of cardiovascular disease [16]. A previous study indicated that leukocyte telomere length is shortened in AS patients [17]. In recent years, it has been found that TERT is widely expressed in macrophages, but its expression and function in AS have not been elaborated well [18]. Moreover, it is found that mitochondria and telomere link contributes to the induction of cancer cell senescence and PGC-1*α* is the key molecule to link oxidative stress and telomere function [19]. Upregulation of PGC-1*α* in aortic smooth muscle cells can enhance the telomere function and reduce DNA damage to inhibit AS [5]. Furthermore, a proper PGC-1*α* activator pioglitazone can prevent stress-induced endothelial apoptosis through activating aortic telomerase [20]. Thus, PGC-1*α* is considered to be an upstream molecule to regulate TERT expression. However, whether TERT is involved in modulating ROS accumulation or apoptosis during AS is largely unknown. Based on the observation that TERT has the potential to be regulated by PGC-1*α*, we hypothesized that a PGC-1*α*/TERT axis might play an important role in regulating telomere function, DNA damage, and ROS accumulation, thus affecting AS.

Age-related telomere shortening may cause DNA breaks, activate the DNA damage response, and trigger cellular senescence, if left unrepaired [21, 22]. p53, whose role is to monitor DNA damage in cells, is a tumor suppressor gene encoding a protein with a molecular weight of 53 kDa. Reports indicated that p53 is involved in TERT-mediated DNA damage, abnormal cell aging, and apoptosis [23, 24]. Thus, p53 was examined as a downstream molecule of TERT and an indicator of DNA damage.

**Figure 1:**
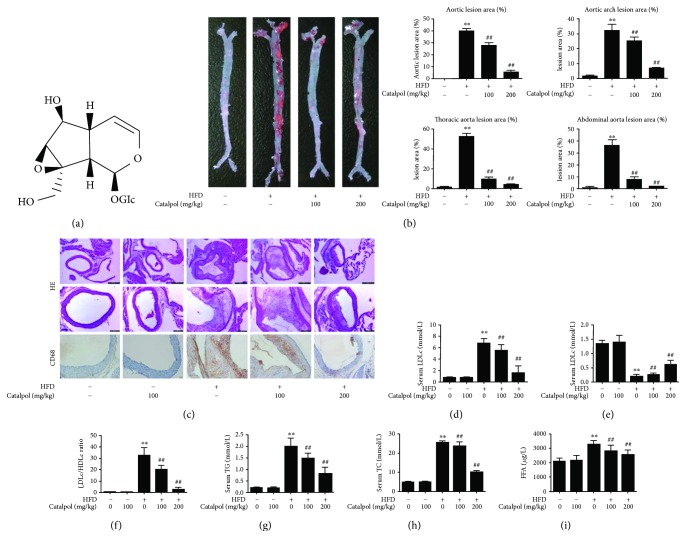
Catalpol ameliorated AS in LDLr^−/−^ mice. 6–8-week-old male LDLr^−/−^ mice were administered with a standard diet with catalpol (0 and 100 mg/kg) or HFD with catalpol (0, 100, and 200 mg/kg). (a) Chemical structure of catalpol. (b) Atherosclerotic lesion formation by red oil O staining, *n* = 10. (c) Atherosclerotic lesion formation by HE staining and macrophage-specific CD68 staining, *n* = 10. (d–f) serum LDL-C and HDL-C assay, *n* = 10. (g–i) serum TG, TC, and FFA assay, *n* = 10. ^∗∗^ *p* < 0.05 compared with the control group, ^##^ *p* < 0.05 compared with the HFD group. Error bars depict the standard deviation. HFD: high-fat diet.

Catalpol is an iridoid glucoside and has been found to be present in large quantities in the root of *Rehmannia glutinosa* L. [25], whose structure is shown in Figure 1(a). To be a traditional medicine, catalpol demonstrates a variety of biological activities including anticancer, neuroprotective, anti-inflammatory, diuretic, hypoglycemic, and antihepatitis virus effects. What is more, some studies have provided some clues that catalpol can affect energy metabolism by increasing mitochondrial biogenesis [26], enhancing endogenous antioxidant enzymatic activities, and inhibiting free radical generation [27]. In our previous study, catalpol produced a strong protective ability against ROS production and apoptosis caused by TNF-*α* in HAECs, and the mechanism might be related to modulating energy metabolism [28]. What is more, catalpol produced lipid-lowering and hypoglycemic effects associated with regulating energy metabolism [25, 29]. In this study, we proposed the hypothesis that catalpol could alleviate AS through the PGC-1*α*/TERT pathway. This will provide new evidence for catalpol in the treatment of AS and throw new light for the complete understanding of the role of the PGC-1*α*/TERT pathway in AS.

## 2. Materials and Methods

### 2.1. Reagents

Catalpol (98%) was obtained from Nanjing Jingzhu Biotech Ltd. Co (Nanjing, Jiangsu, China). The 1640 medium was bought from Gibco-BRL Company (Gaithersburg, MD, USA). A 2′, 7′-dichlorodihydrofluorescein diacetate (H_2_DCFDA) fluorescent probe and ECL Plus were obtained from Beyotime (Nanjing, Jiangsu, China). Antibodies specific for B-cell lymphoma-2 (Bcl-2), Caspase-3, Caspase-9, PGC-1*α*, TERT, and p53 were obtained from Proteintech Group (Wuhan, Hubei, China). An antibody specific for *β*-actin was purchased from Beyotime.

### 2.2. Preparation of oxLDL

Fresh whole blood from a normal human was added with 0.5% EDTA-2Na for anticoagulation. Plasma was isolated by spinning the blood at 4°C and 7000 r/min for 15 min. Native LDL (density: 1.019~1.063 g/mL) was separated from fresh normolipidemic human serum by discontinuous density-gradient ultracentrifugation using a Beckman Coulter Optima L-100 XP Ultracentrifuge and then oxidatively modified [30]. LDL was oxidized with 50 *μ*M CuSO_4_ at 37°C for 24 h and then transferred into EDTA-2Na (200 mmol/L) in phosphate-buffered saline (PBS) for 24 h at 4°C. Subsequently, oxidation was stopped by extensive dialysis against PBS with 0.01% EDTA and sterilized by filtration. LDL oxidation was confirmed by thiobarbituric acid reactive substances with malondialdehyde (MDA) as the standard [31]. LDL and oxLDL protein concentrations were determined with a bicinchoninic acid (BCA) protein assay kit (Beyotime) which used bovine serum albumin as the standard and was expressed as micrograms per milliliter of solution. LDL was prepared every 2 weeks.

### 2.3. Cell Culture and Treatment

Human THP-1 cells were purchased from ScienCell company (CA, USA) and cultured in RPMI 1640 medium (Gibco, CA, USA) with 10% (*v*/*v*) fetal bovine serum (Gibco), 20 mg/mL penicillin, and 20 mg/mL streptomycin and maintained at 37°C in a humidified atmosphere of 5% CO_2_ and then treated with 100 nM phorbol 12-myristate 13-acetate (PMA) (Sigma, St. Louis, Missouri, USA) for 48 h to induce differentiation into macrophages, and then the in vitro model was established by replacing the mediums with a serum-free medium containing oxLDL (100 *μ*g/mL) for 24 h as previously described.

### 2.4. Transfection

THP-1-derived macrophages were transiently transfected using Lipofectamine 2000 (Life Technologies-Invitrogen, Carlsbad, CA, USA) in Opti-MEM according to the manufacturer's protocol, as described [32]. Briefly, THP-1 cells were seeded onto 6-well plates in DMEM, without antibiotics, then 100 nmol PMA for 48 h was added, inducing them to become macrophages; transfection was done with duplex TERT (25 nmol/L), PGC-1*α* (50 nmol/L)-specific siRNAs, and nonspecific (NS) siRNAs, respectively, as described. Cells transfected with NS or specific siRNA for 12 h were treated with oxLDL or catalpol for an additional 24 h, then harvested and analyzed by Western blot analysis. Control nontargeting siRNA, siRNA-targeting TERT (si-TERT, sense: 5′-GGAAGAGUGUCUGGAGCAATTdTdT-3′ and antisense: 5′-UUGCUCCAGACACUCUUCCTT-3′), and siRNA-targeting PGC-1*α* (si-PGC-1*α*, sense: 5′-CCAAGACUCUAGACAACUAdTdT-3′ and antisense: 5′-UAGUUGUCUAGAGUCUUGGdTdT-3′) were obtained from GenePharma Co., Ltd (Shanghai, China).

### 2.5. Cell Viability Assay

THP-1-derived macrophages (8 × 10^3^/mL) were seeded into each of the 96-well culture plates overnight and kept in a humidified atmosphere of 5% CO_2_ and 95% air at 37°C. After 48 h of incubation, the medium was exchanged for serum RPMI 1640 medium and added to catalpol at different concentrations (0, 5, 10, 20, 40, and 80 *μ*M) with or without oxLDL (100 *μ*g/mL) or treated with catalpol for different times (0, 6, 12, 24, and 48 h). Meanwhile, cells without any treatment were used as a control. Following this, the culture medium was removed, and 5 mg/mL methyl thiazolyl tetrazolium (MTT) was added to each well. The plates were then incubated for 4 h at 37°C. The supernatant was carefully removed, the formazan crystals in each well were dissolved in 200 *μ*L of dimethyl sulfoxide (DMSO) for 30 min at 37°C, and optical density at 570 nm was read on a microplate reader (Thermo Fisher Scientific, MA, USA).

### 2.6. Biochemical Analysis

The serum parameters total triglyceride (TG), total cholesterol (TC), and liver tissue free fatty acid (FFA), serum and cell superoxide dismutase (SOD), MDA, lactate dehydrogenase (LDH), and glutathione (GSH) levels were detected using detection kits based on the manufacturer's instructions (Nanjing Jiancheng Institute of Biotechnology, Nanjing, China).

### 2.7. Measurement of Intracellular ROS

An H_2_DCFDA probe was employed to measure the ROS level as previously described [33]. THP-1 macrophages were incubated with oxLDL and different concentrations of catalpol for 24 h at 37°C and then collected and incubated with 20 *μ*M H_2_DCFDA for 30 min at 37°C. Fluorescence intensity was immediately measured using a fluorescent-activated cell sorting (FACS) Calibur flow cytometer (Becton Dickinson Immunocytometry Systems, CA, USA) equipped with an argon ion laser (488 nm excitation), and 20,000 cells per sample were measured.

### 2.8. Luciferase Activity Assay

Luciferase reporter gene vector pGL4 containing the PGC-1*α* promoter area was obtained from Baihao Bio (China, Shenyang). pGL4-PGC-1*α* or control was transfected into THP-1-derived macrophages. Reporter assays were conducted 24 h after administrating with catalpol (0, 5, 20, and 80 *μ*M) or pioglitazone (5 *μ*M). The luciferase activity was determined with a Dual-Luciferase Reporter Assay Kit (Promega, Wisconsin, USA) using a Dual-Light Chemiluminescent Reporter Gene Assay System and was normalized to the Renilla luciferase activity.

### 2.9. Ethics Statement

All experiments were approved by the Animal Care and Use Committee of Dalian Medical University, and the experimental procedures were performed in strict accordance with legislation regarding the use and care of laboratory animals of China.

### 2.10. Animals and Diets

Male eight-week-old LDL receptor knockout (LDLr^−/−^) mice with a C57BL/6 background were purchased from Vital River Laboratory Animal Technology Co., Ltd. (Beijing, China). Before the experiments, the animals were allowed to suit the new environment for 7 days and housed in a room under a 12 h light/dark cycle, a controlled temperature at 22 ± 3°C, and a relative humidity at 60 ± 10%. To detect the effect of catalpol on atherosclerosis, sixty LDLr^−/−^ mice were divided into five groups: chow diet (*n* = 12), catalpol (100 mg/kg/D, *n* = 12), high-fat diet (HFD, *n* = 12), HFD with catalpol (100 mg/kg/D, *n* = 12), and HFD with catalpol (200 mg/kg/D, *n* = 12) for 16 weeks. The diet was a commercially prepared mouse food (MD12017) supplemented with 20.0% (wt/wt) cocofat, 1.25% (wt/wt) cholesterol, and 22.5% (wt/wt) protein and 45.0% carbohydrate (Jiangsu Medicience Ltd., Jiangsu, China). At week 16, mice were anesthetized with 2% isoflurane (Forene®, Abbott), one milliliter of blood was collected by abdominal aorta, and tissues were collected for further analysis.

### 2.11. Lesion Assessment by Oil Red O Staining

The aortic arch and thoracic and abdominal aortae were cut longitudinally and pinned flat. Tissues were rinsed in PBS and 60% isopropanol and stained in oil red O. *En face* lesion size was calculated as a ratio of oil red O positive staining to the total surface area.

### 2.12. Hematoxylin-Eosin (HE) Staining

The upper half of the heart that contained the aortic origin was fixed with 10% buffered formalin solution for 30 min and then dehydrated in 75% ethanol overnight, followed by paraffin embedding. For morphometric analysis of atherosclerotic lesions, serial 4 *μ*m sections were cut. The sections were stained with hematoxylin and eosin for histologic analysis.

### 2.13. Immunohistochemical (IHC) Analyses

Paraffin sections (4 *μ*m) of the aortic root were used for immunohistochemistry analysis. IHC staining was undertaken using a rabbit polyclonal antibody to CD68 (1 : 100 dilution; Proteintech). Immune activity in the sections was identified by reactions with a biotinylated secondary antibody (1 : 200, 1 h at 37°C) and streptavidin-biotin-peroxidase (1 : 200, 1 h at 37°C), followed by counterstaining with hematoxylin, dehydration, and mounting. Images were acquired and processed in TIFF format using Image-Pro Plus® (Media Cybernetics).

### 2.14. *β*-Galactosidase Staining

THP-1-derived macrophages treated with catalpol either alone or in the presence of oxLDL were stained with the senescence-associated *β*-galactosidase staining solution (Beyotime) following the manufacturer's instructions.

### 2.15. Western Blot Analysis

Cells and tissues were harvested and protein extracts prepared according to established methods. Extracts were separated in sodium dodecyl sulfate-polyacrylamide electrophoresis gels (8~15%) and transferred to a polyvinylidene difluoride (PVDF) membrane (Millipore, Bedford, MA, USA). The membranes were blocked with 5% milk and then incubated with indicated primary antibodies at 4°C overnight. After washing, the membranes were then incubated with the appropriate secondary antibodies. The membranes were exposed to enhanced chemiluminescence plus reagents (Beyotime). The emitted light was captured by a Bio-Rad imaging system with a Chemi HR camera 410 and analyzed with a Gel-Pro Analyzer Version 4.0 (Media Cybernetics, MD, USA).

### 2.16. TUNEL Staining

Apoptosis was quantified in paraffin-embedded aorta using a terminal deoxynucleotidyl transferase-mediated deoxyuridine triphosphate nick-end labeling (TUNEL) Assay kit (TUNEL Apo-Green Detection Kit, Biotool, Houston, USA) according to the manufacturer's instructions. Green fluorescence staining indicated positive TUNEL staining.

### 2.17. Comet Assay

The comet assay was used to identify the mechanisms behind the protective effect of catalpol. This assay was performed according to the manufacturer's instructions (KeyGen Biotech, China). THP-1 cells were seeded in 6-well plates and treated with PMA (100 nM) for 48 h and oxLDL or catalpol or both. After 24 h, cells were harvested and the cell suspension was mixed with 75 *μ*L low melting agarose in a density of 2 × 10^4^ cells/mL and directly pipetted on agarose-projected slides. Slides were stored at 4°C for 30 min and subsequently submerged in lysis solution. After 60 min lysis, they were treated with alkaline unbending solution (pH > 13) for 60 min, followed by 30 min electrophoresis at 25 V. Slides were stained with PI and visualized and photographed by a digital camera attached to a fluorescent microscope using a 20x magnification.

### 2.18. 8-Hydroxydeoxyguanosine (8-OHdG) Analysis

DNA was isolated according to the protocol's instruction, and the RNA-free DNA obtained was used to determine 8-OHdG levels using the 8-OHdG ELISA kit (Huamei, Wuhan, China) according to the manufacturer's instructions.

### 2.19. Determination of Telomerase Activity

Telomerase activity was determined by telomeric repeat amplification protocol (TRAP) assay [34]. Briefly, cell pellets were lysed in CHAPS lysis buffer containing a ribonuclease inhibitor and incubated for 30 min at 4°C. Cell extract (1 mg) was mixed with a TRAPeze reaction mix (KeyGen Biotech, Jiangsu, China) containing telomerase substrate (TS) primer, GAPDH primer, and Maxima SYBR Green qPCR Master Mix. Extension of TS primers was performed at 30°C for 30 min followed by PCR amplification. TRAP products from the same experiment were analyzed for quantification of telomerase activity by the 2^−ΔΔCT^ method. Means ± SD were calculated from three independent assays.

### 2.20. Statistical Analysis

All results are expressed as the mean ± standard deviation (SD) from more than three independent experiments, and data analyses were performed with the SPSS software package, version 19.0. Comparison of quantitative variables was performed by either Student's *t*-test or analysis of variance (ANOVA) followed by the Student-Newman-Keuls (SNK) test. *p* values < 0.05 (two-tailed) were considered statistically significant.

## 3. Results

### 3.1. Catalpol Inhibited the Development of Atherosclerotic Lesions and Improved Lipid Profiles

In order to elucidate the effect of catalpol on the development of AS, aortic lesions were measured by oil red O staining. The result showed that the lesions were grossly larger and thicker in HFD mice than those in the control mice; however, catalpol could reduce the lesion area and the thickness in the whole aorta especially in the aortic arch (Figure 1(b)). Then, the intimal lesions were further evaluated under microscopy, as shown in Figure 1(c). HE staining indicated that the aortas from the HFD group exhibited an increased intimal lesion area containing a necrotic core. Moreover, immunohistochemical staining showed that the necrotic core was enriched in macrophage-derived foam cells. In the tunic media, smooth muscle cells exhibited a changed shape, going from a concentric circular configuration to a more radial shape. On the contrary, in the group treated with catalpol, there were only a smaller number of foam cells in the subendothelial space with the preservation of the tunic media, especially in the high-dose group of catalpol (200 mg/kg). These results indicated that catalpol could inhibit the development of atherosclerotic lesions.

The serum levels of TC, LDLc, HDLc, TG, and FFA were determined to investigate the effect of catalpol on lipid profiles. As shown in Figures 1(d) and 1(g)–1(i), serum TC, TG, LDLc, and FFA levels in the HFD group were significantly increased. However, in the groups fed with catalpol, these levels were decreased markedly compared with the HFD group. As shown in Figure 1(e), the HDLc level in the HFD group was decreased; however, catalpol could increase the HDLc level obviously compared with the HFD group. As a result, the LDLc/HDLc ratio was significantly decreased compared with the HFD group, as shown in Figure 1(f). The above result indicated that catalpol could improve lipid profiles in LDLr^−/−^ mice.

### 3.2. Catalpol Increased SOD and GSH Content and Inhibited MDA Content and LDH Release

**Figure 2:**
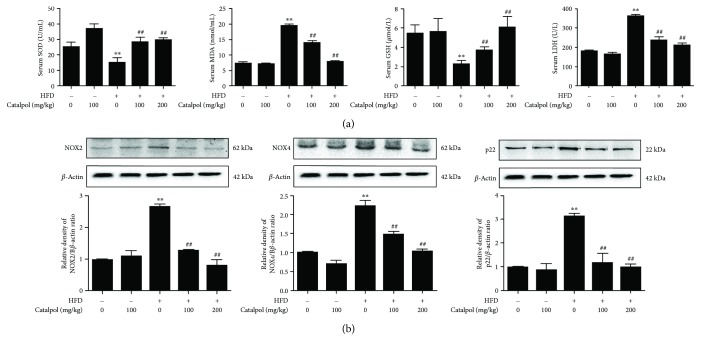
Catalpol reduced oxidative stress in AS. 6–8-week-old male LDLr^−/−^ mice were administered with a standard diet with catalpol (0 and 100 mg/kg) or HFD with catalpol (0, 100, and 200 mg/kg). (a) Effect of catalpol on serum GSH, SOD, and MDA contents and LDH release, *n* = 10. (b) Effect of catalpol on NOX2, NOX4, and p22 protein expression in HFD-treated LDLr^−/−^ mice, *n* = 10. ^∗∗^ *p* < 0.05 compared to the control group; ^##^ *p* < 0.05 compared to the HFD group. Error bars depict the standard deviation. HFD: high fat diet.

Oxidative stress has been considered as an important factor in the pathophysiology of AS [35], and agents with antioxidant activity have a potential role in preventing AS. To further investigate the antioxidant activity of catalpol, activities of SOD, MDA, and GSH and the level of LDH release were determined in vivo and in vitro. As shown in Figure 2(a), serum levels of SOD and GSH were significantly decreased in the HFD group compared to the control group, and in mice fed with catalpol, the levels were significantly increased compared to HFD mice. However, serum LDH and MDA levels were obviously increased in the HFD group compared with the control group, and in mice fed with catalpol, the levels were dramatically decreased compared to HFD mice. These results indicated the potential antioxidant effect of catalpol in LDLr^−/−^ mice.

To verify the antioxidant effect of catalpol, oxLDL-treated macrophages were also employed in an in vitro study. Concentrations of oxLDL and catalpol were determined by MTT assay which are shown in Supplementary Figure S1A; oxLDL (100 *μ*g/mL) and catalpol (5, 20, and 80 *μ*M) were chosen for the following experiments. Then, we examined whether catalpol produced effects on levels of SOD, MDA, GSH, and LDH in oxLDL-treated macrophages. As shown in Supplementary Figure S1B, oxLDL treatment significantly decreased GSH and SOD activity and increased MDA and LDH release in oxLDL-treated macrophages compared to the control group. On the contrary, catalpol apparently reversed the effects of oxLDL in a concentration-dependent manner. Thus, the above results indicated that catalpol could produce antioxidative effects both in HFD-treated LDLr^−/−^ mice and in oxLDL-treated macrophages.

### 3.3. Catalpol Inhibited NOX2 and NOX4 Protein Expression and ROS Accumulation

The nicotinamide adenine dinucleotide phosphate-oxidase (NADPH oxidase) family consisting of NOX1–5 is the most important source of ROS. NOX2 and NOX4 are major sources of ROS in macrophages. In addition, p22phox is a critical component of the superoxide-generating NADH/NADPH oxidase system [36]. Thus, p22, NOX2, and NOX4 protein expression and ROS production were determined. As shown in Figure 2(b), protein expressions of p22, NOX2, and NOX4 were significantly increased in HFD-treated LDLr^−/−^ mice compared with the control group, and catalpol significantly reduced these protein expressions. The effect of catalpol on NOX2 and NOX4 protein expression was also recapitulated in oxLDL-treated macrophages (Supplementary Figure S1C). Further, ROS production in oxLDL-treated macrophages was detected and increased ROS production was found in oxLDL-treated macrophages compared with the control group, and catalpol significantly inhibited the level of ROS production compared with the oxLDL group (Supplementary Figure S1D). The above results indicated a potential role of catalpol in inhibiting oxidative stress both in AS mice and in oxLDL-treated macrophages.

### 3.4. Catalpol Inhibited DNA Oxidative Damage and Cell Apoptosis

**Figure 3:**
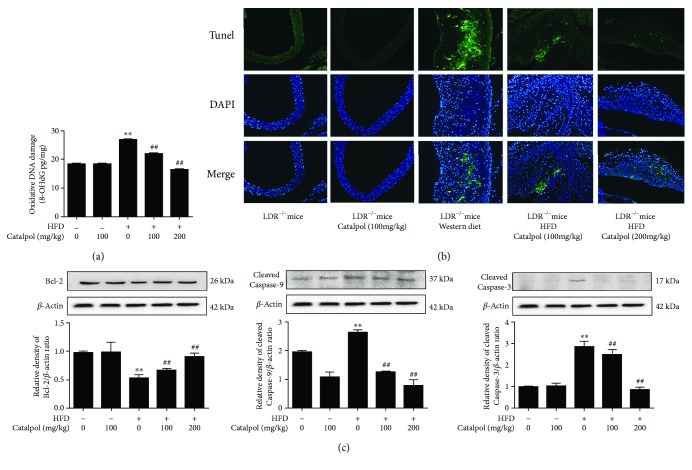
Catalpol inhibited DNA oxidative damage and cell apoptosis. 6–8-week-old male LDLr^−/−^ mice were administered with a standard diet with catalpol (0 and 100 mg/kg) or HFD with catalpol (0, 100, and 200 mg/kg). (a) Catalpol inhibited oxidative DNA damage in HFD-treated LDLr^−/−^ mice, *n* = 10. (b) Catalpol inhibited cell apoptosis in HFD-treated LDLr^−/−^ mice, *n* = 10. (c) Catalpol inhibited cleaved Caspase-3 and Caspase-9 expression and increased Bcl-2 protein expression in HFD-treated LDLr^−/−^ mice, *n* = 10. ^∗∗^ *p* < 0.05 compared to the control group; ^##^ *p* < 0.05 compared to the HFD group. Error bars depict the standard deviation. HFD: high-fat diet.

High level of ROS production is a well-known cause of DNA oxidative damage [37]. In this study, 8-OHdG level was measured to evaluate the oxidative DNA damage level. As shown in Figure 3(a), the 8-OHdG level was significantly increased in HFD-treated LDLr^−/−^ mice compared to the control mice, and catalpol treatment significantly reduced the 8-OHdG level in a concentration-dependent manner. An increased 8-OHdG level was also found in oxLDL-treated macrophages, and catalpol decreased the increased 8-OHdG level induced by oxLDL (Supplementary Figure S2A). Further, to verify the effect of catalpol on inhibiting DNA damage, comet assay was employed to detect it. The length of comet tailing is proportional to the severity of DNA damage. As shown in Supplementary Figure S2B, oxLDL treatment significantly increased the length of comet tailing. However, catalpol treatment partly inhibited the increased DNA damage level induced by oxLDL. High level of ROS is also an important cause to induce cell apoptosis. It is commonly believed that the necrotic core in AS plaque is formed when lipid-laden macrophages die through either apoptosis or necrosis and deposits cellular debris as well as lipid material in the core of the plaque [38]. Thus, TUNEL staining which can stain apoptotic cells in green was employed to detect apoptosis. As shown in Figure 3(b), in HFD LDLr^−/−^ mice, increased apoptotic or necrotic cells were detected in the plaque area compared with the control mice, but administration with catalpol largely reduced the plaque area and TUNEL-positive cells compared with the HFD group. In Figure 3(c), Western blotting analysis showed a decreased Bcl-2 and an increased cleaved Caspase-3 and -9 protein expression levels in the HFD group compared with the control group; however, catalpol significantly reversed these effects induced by HFD. Similar effects of catalpol were also observed in oxLDL-treated cells, which are shown in Supplementary Figures S2C and S2D. Collectively, these results indicated that catalpol could produce a potential antiapoptotic effect which might contribute to inhibiting AS.

### 3.5. Catalpol Relieved Cellular Senescence and Telomere Dysfunction

**Figure 4:**
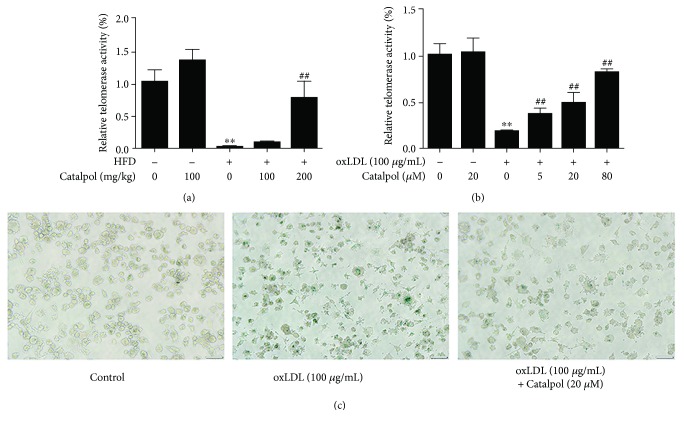
Catalpol increased telomerase activity and inhibited macrophage senescence. 6–8-week-old male LDLr^−/−^ mice were administered with a standard diet with catalpol (0 and 100 mg/kg) or HFD with catalpol (0, 100, and 200 mg/kg). THP-1 cells were exposed to PMA (100 ng/mL) for 72 h to induce macrophage formation. Then, macrophages were treated with oxLDL or catalpol (0, 5, 20, and 80 *μ*M) for 24 h as indicated. Catalpol increased telomerase activity in (a) LDLr^−/−^ mice and (b) oxLDL-treated macrophages, *n* = 10. ^∗∗^ *p* < 0.05 compared to the control group; ^##^ *p* < 0.05 compared to the HFD group. Error bars depict the standard deviation. (c) Catalpol inhibited macrophage senescence by *β*-galactosidase staining. HFD: high-fat diet.

Telomere dysfunction and shortening are important features of cellular senescence in AS. Thus, in this study, telomere activity and cell senescence were measured by TRAP assay and *β*-galactosidase staining. As in data shown in Figure 4(a), HFD significantly reduced telomerase activity compared with the control group and catalpol partly restored the decreased telomerase activity compared with the HFD group. Moreover, there is also a slight increase in telomerase activity in the group given catalpol only compared with the control group. In the in vitro study, decreased telomerase was found in oxLDL-treated macrophages compared with the control group and catalpol could obviously increase the telomerase activities in a concentration-dependent manner, as shown in Figure 4(b). As expected, increased cell senescence in oxLDL-treated macrophages was found compared to the control group by *β*-galactosidase staining. And catalpol partly inhibited the macrophage senescence compared to the oxLDL group (as shown in Figure 4(c)). The above findings indicated the potential role of catalpol on restoring telomerase activities and inhibiting cell senescence.

### 3.6. Activation of the PGC-1 *α*/TERT Pathway Was Involved in the Protective Effect of Catalpol against AS

**Figure 5:**
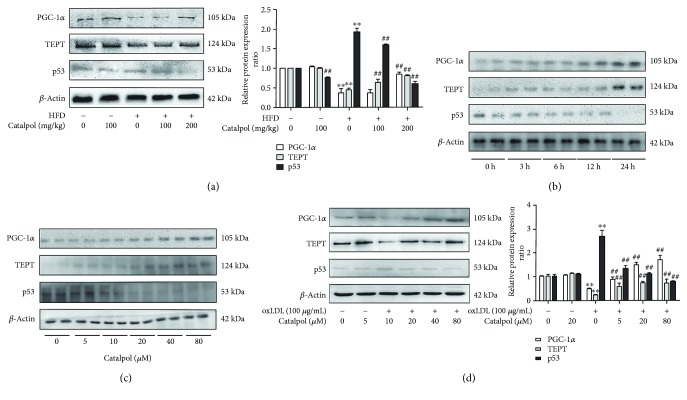
Activation of the PGC-1 *α*/TERT pathway was involved in the protective effect of catalpol against AS. 6–8-week-old male LDLr^−/−^ mice were administered with a standard diet with catalpol (0 and 100 mg/kg) or HFD with catalpol (0, 100, and 200 mg/kg). (a) Catalpol increased PGC-1*α* and TERT expression and decreased p53 expression in HFD-treated LDLr^−/−^ mice, *n* = 10. (b) THP-1 cells were exposed to PMA (100 ng/mL) for 72 h to induce macrophage formation. Then, macrophages were treated with catalpol (80 *μ*M) for 0, 3, 6, 12, and 24 h. Catalpol increased PGC-1*α* and TERT expression and decreased p53 expression in a time-dependent manner, *n* = 10. (c) THP-1 cells were exposed to PMA (100 ng/mL) for 72 h to induce macrophage formation. Then, macrophages were treated with catalpol (0, 5, 10, 20, 40, and 80 *μ*M) for 24 h. Catalpol increased PGC-1*α* and TERT expression and decreased p53 expression in a concentration-dependent manner, *n* = 10. (d) THP-1 cells were exposed to PMA (100 ng/mL) for 72 h to induce macrophage formation. Then, macrophages were treated with oxLDL (0 and 100 *μ*g/mL) or catalpol (0, 5, 20, and 80 *μ*M) for 24 h. Catalpol increased PGC-1*α* and TERT expression and decreased p53 expression in oxLDL-treated macrophages, *n* = 10. ^∗∗^ *p* < 0.05 compared to the control group; ^##^ *p* < 0.05 compared to the HFD group. Error bars depict the standard deviation. HFD: high fat diet.

To address the issue of whether catalpol protects against AS by upregulating the PGC-1 *α*/TERT pathway, we measured the effects of catalpol on the protein expressions of PGC-1*α*, TERT, and p53 both in vivo and in vitro. As shown in Figure 5(a), after 16-week HFD, the expression of PGC-1*α* was decreased to 37%, and TERT protein expression was decreased to 45% in LDLr^−/−^ mice; however, the p53 level was increased to 1.92-fold compared to that in the control group. However, mice fed up with catalpol showed significantly upregulated PGC-1*α* and TERT protein expressions and decreased p53 protein expression indicating that activation of the PGC-1*α*/TERT pathway might be involved in AS progress and catalpol's effect against AS.

We next determined whether the in vivo results could be recapitulated in oxLDL-treated macrophages. At first, effects of catalpol on PGC-1*α*, TERT, and p53 protein levels were detected after treatment with catalpol. As shown in Figure 5(b), catalpol (80 *μ*M) upregulated PGC-1*α* and TERT protein levels at 3 h after administration and had a significant increase by 2.72- or 2.14-fold at 12 h and 7.14- or 4.57-fold at 24 h. However, p53 protein expression was in an opposite profile. Then, macrophages were incubated with catalpol (0, 5, 10, 20, 40, and 80 *μ*M) for 24 h and PGC-1*α*, TERT, and p53 protein expressions were detected by Western blotting. And the result showed that catalpol increased PGC-1*α* and TERT protein expressions and decreased p53 protein expression in a concentration-dependent manner (Figure 5(c)). As shown in Figure 5(d), in oxLDL-treated macrophages, PGC-1*α* and TERT protein levels were significantly decreased and p53 protein level was significantly increased compared with those in the control group, and catalpol reversed the decrease in PGC-1*α* and TERT expression and increase in p53 protein expressions, which was consistent with the results in LDLr^−/−^ mice. Collectively, our study demonstrated that PGC-1*α* decrease might be a critical pathogenic mechanism during AS and catalpol might protect against AS through activating the PGC-1*α*/TERT pathway.

### 3.7. Catalpol Decreases ROS Accumulation, DNA Damage and Ameliorates Telomere Function through Upregulating PGC-1*α* Expression

**Figure 6:**
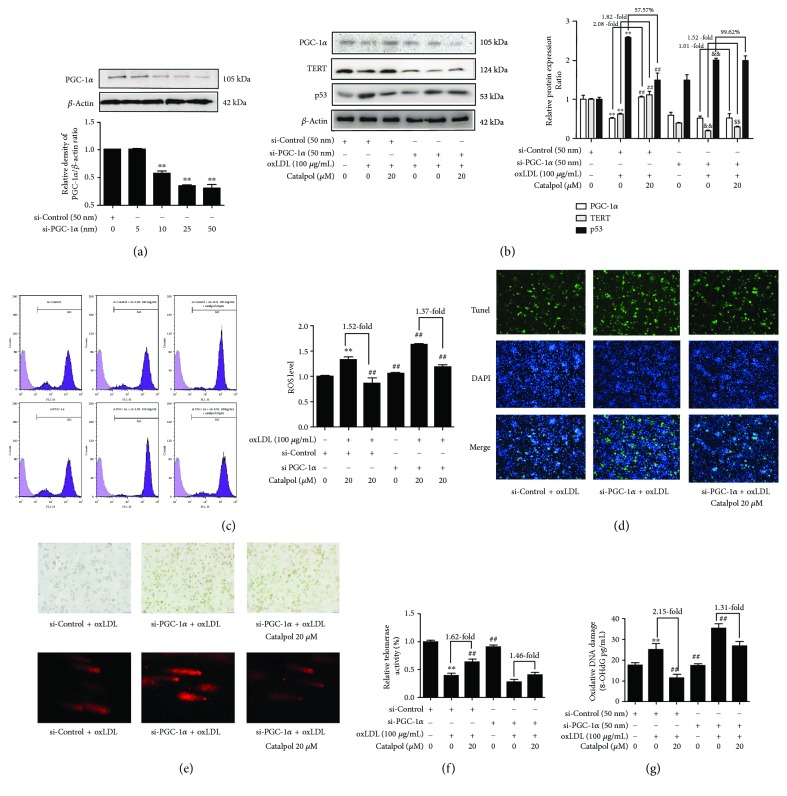
Catalpol decreases ROS accumulation and DNA damage and ameliorates telomere function through upregulating PGC-1*α* expression. THP-1 cells were exposed to PMA (100 ng/mL) for 72 h to induce macrophage formation and then transfected with small nontargeting RNA for si-control or si-PGC-1*α* for 12 h, then treated with oxLDL or catalpol for an additional 24 h and harvested and analyzed by Western blot analysis. (a) Knockdown of PGC-1*α* by its specific siRNA, *n* = 10. (b) Effect of catalpol on increasing PGC-1*α* and TERT protein expression and decreasing p53 protein expression was attenuated with PGC-1*α* siRNA, *n* = 10. (c) Effect of catalpol on ROS production with PGC-1*α* siRNA, *n* = 10. (d) Effects of catalpol on cell apoptosis with PGC-1*α* siRNA, *n* = 10. (e) Effects of catalpol on cell senescence and DNA damage, *n* = 10. (f) Telomerase activity, *n* = 10. (g) Oxidative DNA damage, *n* = 10. ^∗∗^ *p* < 0.05 compared to the control group; ^##^ *p* < 0.05 compared to the oxLDL group; ^&&^ *p* < 0.05 compared with the si-PGC-1*α* group, ^$$^ *p* < 0.05 compared with the oxLDL-treated si-PGC-1*α* group. Error bars depict the standard deviation.

To evaluate the potential roles of PGC-1*α* in the effect of catalpol inhibiting AS, we transfected PGC-1*α* siRNA in oxLDL-treated macrophages. As shown in Figure 6(a), transfected PGC-1*α* siRNA (50 nm) potently inhibited PGC-1*α* protein expression level to 30.51% compared with the si-control group, indicating that PGC-1*α* expression was knocked down successfully. After being transfected with PGC-1*α* siRNA, the basal TERT expression was reduced by 40.51% but the basal p53 expression was increased by 1.47-fold. In addition, the upregulating effect of catalpol on PGC-1*α* and TERT and the downregulating effect on p53 were attenuated (Figure 6(b)). Furthermore, PGC-1*α* knockdown profoundly promoted ROS production and attenuated the inhibitory effect of catalpol on ROS production from 1.52-fold to 1.37-fold compared with that in normal cells (Figure 6(c)). As shown in Figure 6(d), PGC-1*α* siRNA transfection significantly increased cell apoptosis and decreased the inhibitory effect of catalpol. As shown in Figure 6(e), PGC-1*α* siRNA transfection promoted cell senescence and increased the comet tail length and also attenuated the protective effect of catalpol. Moreover, PGC-1*α* siRNA transfection significantly reduced telomerase activity (Figure 6(f)) and upgraded the oxidative DNA damage level (Figure 6(g)). The promoting effect of catalpol on telomerase activity was also attenuated from 1.62-fold to 1.46-fold, and the inhibitory effect of catalpol on DNA oxidative damage was attenuated from 2.15-fold to 1.31-fold. These data indicated that catalpol induced protective effects in AS possibly through upregulating PGC-1*α*.

### 3.8. TERT Upregulation Was Involved in the Protective Effect of Catalpol on ROS Accumulation, DNA Damage, and Telomere Dysfunction

To further investigate the potential roles of TERT and the underlying mechanisms of catalpol in inhibiting AS, TERT siRNA was also employed in this investigation. As shown in Supplementary Figure S3A, TERT siRNA 25 nM showed effectively in inhibiting TERT expression and was used in the following study. As shown in Supplementary Figure S3B, knockdown of TERT expression by its specific siRNA could induce a decrease in PGC-1*α* basal expression level but evoked an increase in basal p53 protein expression. Although basal PGC-1*α* expression was decreased by TERT siRNA, the upregulating effect of catalpol on PGC-1*α* expression did not change much (1.84-fold and 1.90-fold). Thus, we suggested that catalpol upregulated PGC-1*α* protein expression independent of TERT. Moreover, the inhibitory effect of catalpol on p53 protein expression was attenuated by TERT siRNA. The results indicated that there is a crosstalk between PGC-1*α* and TERT, and p53 protein expression is partly regulated by PGC-1*α* and TERT. Furthermore, TERT knockdown profoundly promoted ROS production (Supplementary Figure S3C) and cell senescence and increased the comet tail length (Supplementary Figure S3E). And TERT siRNA transfection significantly reduced the protective effect of catalpol. As shown in Supplementary Figure S3D, TERT siRNA transfection promoted cell apoptosis and attenuated the inhibitory effect of catalpol as expected. Moreover, TERT siRNA significantly reduced telomerase activity (Supplementary Figure S3F) and increased the oxidative DNA damage level (Supplementary Figure S3G). Similarly, after transfection with TERT siRNA, the protective effects of catalpol on these events were attenuated compared with those in the normal cells. These results demonstrated that catalpol produced these protective effects partly via upregulating TERT. Compared with these results in the experiment with PGC-1*α* siRNA, we found that the regulation on DNA damage and telomerase activity is more dependent on TERT; however, ROS accumulation is much more dependent on PGC-1*α*.

### 3.9. Correlation between PGC-1*α* and TERT in Catalpol-Mediated ROS Accumulation, DNA Damage, and Telomere Function

**Figure 7:**
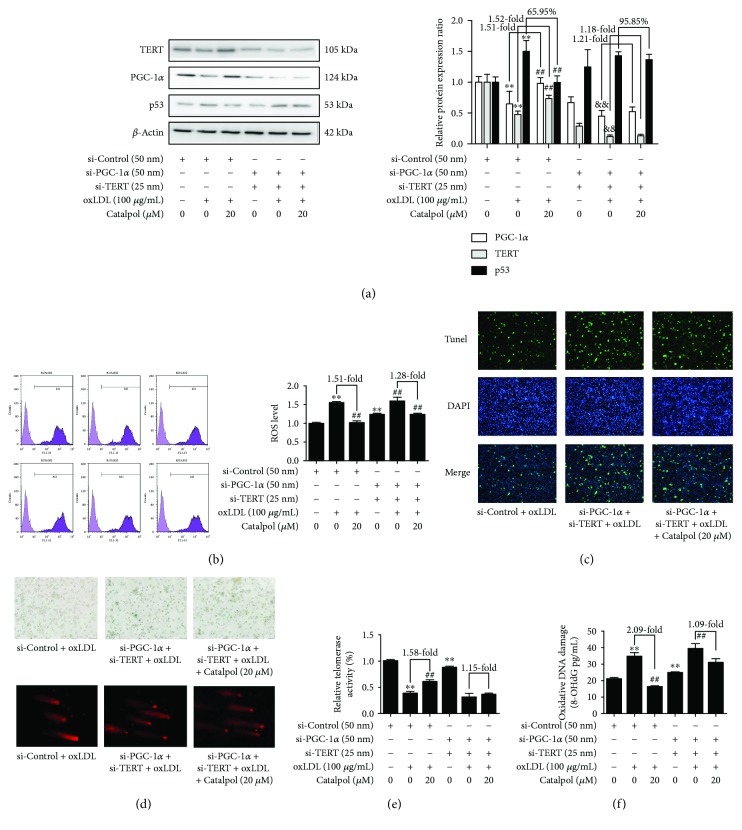
Catalpol decreases DNA damage and ROS accumulation and ameliorates telomere function in THP-1 macrophages through the PGC-1*α*/TERT pathway. THP-1 cells were exposed to PMA (100 ng/mL) for 72 h to induce macrophage formation and then transfected with small nontargeting RNA for si-control or si-TERT and si-PGC-1*α* for 12 h, then treated with ox-LDL or catalpol for an additional 24 h and harvested and analyzed by Western blot analysis. (a) Effect of catalpol on PGC-1*α* and TERT protein expression and decreasing p53 protein expression with PGC-1*α* and TERT siRNA, *n* = 10. (b) Effect of catalpol on ROS production with PGC-1*α* and TERT siRNA, *n* = 10. (c) Effect of catalpol on cell apoptosis with PGC-1*α* and TERT siRNA, *n* = 10. (d) Effects of catalpol on cell senescence and DNA damage, *n* = 10. (e) Telomerase activity, *n* = 10. (f) oxidative DNA damage, *n* = 10.^∗∗^ *p* < 0.05 compared to the control group; ^##^ *p* < 0.05 compared to the oxLDL group; ^&&^ *p* < 0.05 compared with the si-PGC-1*α* and TERT groups. Error bars depict the standard deviation.

Double knockdown of PGC-1*α* and TERT by their specific siRNA was also used to verify the regulatory effect of catalpol on the PGC-1*α*/TERT pathway and the relation between PGC-1*α* and TERT. Reduced protein expressions of PGC-1*α* and TERT after transfection of siRNA are shown in Figure 7(a). After double knockdown of PGC-1*α* and TERT, the basal p53 protein expression level was significantly increased, and the inhibitory effect of catalpol on p53 protein expression almost completely disappeared (Figure 7(a)). This result indicated that the p53 protein expression level is negatively regulated by both PGC-1*α* and TERT. As expected, a more significantly increased ROS production (Figure 7(b)), cell senescence, and the comet tail length (Figure 7(d)) were detected compared to PGC-1*α* or TERT knockdown alone. A significantly increased cell apoptosis (Figure 7(c)), reduced telomerase activity (Figure 7(e)), and increased oxidative DNA damage level (Figure 7(f) were also detected, and the effect of catalpol was much attenuated compared to PGC-1*α* or TERT knockdown alone. Collectively, these data verified that catalpol evoked such protective effects via the PGC-1*α*/TERT pathway.

### 3.10. Catalpol Directly Enhances PGC-1*α* Promoter Activity

**Figure 8:**
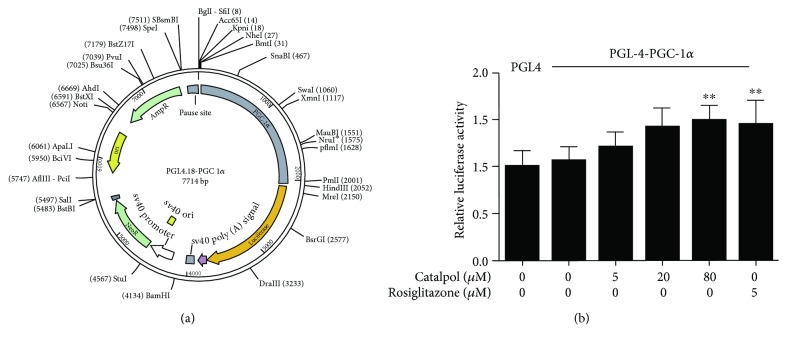
Catalpol directly enhances PGC-1*α* promoter activity. Luciferase reporter gene vector pGL4 containing the PGC-1*α* promoter area was obtained and transfected into THP-1-derived macrophages. Reporter assays were conducted 24 h after administrating with catalpol (0, 5, 20, and 80 *μ*M) or pioglitazone (5 *μ*M). The luciferase activity was determined and was normalized for Renilla luciferase activity. (a) The PGC-1*α* promoter was inserted into the PGL4 vector. (b) Effect of catalpol on PGC-1*α* promoter activity by dual luciferase activity, *n* = 10. ^∗∗^ *p* < 0.05 compared to the PGL4 group. Error bars depict the standard deviation.

Based on the above results that TERT knockdown did not attenuate the increasing effect of catalpol on PGC-1*α*, we speculate that catalpol performed such effects through directly upregulating PGC-1*α* but not TERT; thus, we detected the effect of catalpol on the PGC-1*α* promoter by luciferase activity assay. The PGC-1*α* promoter was inserted into the PGL4 vector as shown in Figure 8(a). In this experiment, a potent agonist of PGC-1*α*, rosiglitazone (5 *μ*M), was employed as a positive control (Figure 8(b)). As expected, catalpol (80 *μ*M) significantly increased luciferase activity by 1.49-fold compared with the PGL4-PGC-1*α* control group, and the efficacy is similar with rosiglitazone (5 *μ*M). The result indicated a potential effect of catalpol on enhancing PGC-1*α* promoter activity.

## 4. Discussion

AS has been recognized as a chronic degenerative disease closely related with aging. The use of natural antioxidants affect AS, which has been implicated to be of great clinical relevance [39]. Catalpol is a natural product present in the root of *Rehmannia glutinosa* with multiple pharmacological effects, and recent studies indicated antioxidant and antiaging effects of catalpol [28]. This study was designed to examine the protective effects of catalpol on atherosclerosis and the probable signal pathways.

The aging rate has been associated with the production of high levels of ROS [40] which was considered to be a novel independent risk factor for spontaneous AS [41]. PGC-1*α* is a positive and direct regulator inhibiting ROS production [42, 43] and a master regulator of cellular energy metabolism [44]. Loss of PGC-1*α* expression was found in clinical and experimental AS models, and upregulation of PGC-1*α* is being reported to be antiatherogenic [45]. In our study, we demonstrated a decrease of PGC-1*α* in both in vivo and in vitro AS models, and upregulating PGC-1*α* protein expression by catalpol attenuated AS plaque formation and cell oxidative stress. Moreover, a previous study has proposed that NOX1 activation can lead to cellular senescence of VSMCs through decreasing PGC-1*α* and increasing mitochondrial oxidative stress [46]. Some natural products have been reported to confer protective effects against AS through the PGC-1*α*-related pathway [47–51]. Likewise, in the present study, catalpol effectively increased the suppression of PGC-1*α* and inhibited the activation of NOX2 and NOX4, as well as ROS overproduction both in vivo and in vitro. Moreover, by using PGC-1*α* siRNA-transfected cells, we confirmed the inhibitory effect of catalpol on ROS production which was partly mediated by increasing PGC-1*α* protein expression.

Telomeres are gene sequences present at chromosomal ends and are responsible for maintaining genome integrity. Telomere length is considered as a biomarker of chronological aging [39]. TERT is a key enzyme in regulating telomere length. Previous reports indicated that interindividual variation in leukocyte telomere length is associated with susceptibility to cardiovascular disease. Some researches also indicated that leukocyte telomere length is shortened in patients with clinical AS [17]. Alternatively, it has also been reported that shorter telomere length may independently serve as a potential predictor of peripheral arterial disease [52]. In our study, loss of TERT protein expression and decreased telomere activity were detected in AS mice and oxLDL-treated macrophages. Catalpol could partly reverse these changes which indicated a potential effect to reduce vascular aging.

Excessive ROS production may lead to a consequence of telomere shortening [53] and induce DNA oxidative stress and cell apoptosis. A previous survey showed a positive relation between oxidative stress and leukocyte telomere length in clinical patients with premature coronary artery [54]. In our study, high levels of ROS production were determined in AS mice and oxLDL-treated macrophages. In addition, by using TERT-specific siRNA, we found that TERT could negatively regulate ROS production and cell apoptosis. Catalpol produced such protective effects depending on enhancing TERT protein expression.

TERT is regarded as a potential target to prevent vascular aging. However, the mechanisms regulating TERT in AS have not yet been determined. The linkage between mitochondrial and telomere length has been proposed in aged people [55]. Researches in primary T cells also indicated that mitochondrial stress contributed to telomere attrition [56]. PGC-1*α* and TERT are two important molecules existing in mitochondria and telomere, respectively. A previous study proposed a pivotal role for PGC-1*α* in maintaining TERT expression in smooth muscle cells of mice [10], which is based on the mitochondria and telomere link. However, the relationship between PGC-1*α* and TERT in macrophages is rarely reported. In the current study, decreased TERT protein expression in AS mice and oxLDL-treated macrophages was demonstrated. Crosstalk between PGC-1*α* and TERT was validated by using PGC-1*α* siRNA and TERT siRNA. Moreover, in our study, inhibited PGC-1*α* and TERT expression was upregulated by catalpol both in vivo and in vitro. It is notable that the upregulating effect of catalpol on PGC-1*α* did not change much after TERT knockdown indicating that PGC-1*α* may be the probable target of catalpol. By using luciferase activity assay, we verified that catalpol could activate the PGC-1*α*/TERT pathway through enhancing PGC-1*α* promoter activity.

Oxidative stress, DNA damage, and telomere shortening are important features of senescent cells. In the current study, ROS production, DNA oxidative damage level, and apoptotic cell numbers are increased in AS mice and oxLDL-treated macrophages, whereas telomere activity showed the opposite pattern. These changes were partly reversed by catalpol indicating the atheroprotective effects of catalpol. P53 is known as a tumor suppressor gene, which plays critical roles in DNA repair and cell apoptosis [57]. Moreover, it regulates the chronic type of stress associated with aging. Indeed, decreased gene dosage of p53 displays elongated longevity and delayed aging [58]. Previous studies indicated that TERT suppression-induced DNA damage and apoptosis was in a p53-dependent way [59]. In the present study, we observed an increase in p53 protein expression and DNA damage level after PGC-1*α* or TERT knockdown. These results confirmed that the PGC-1*α*/TERT pathway mediated DNA damage, apoptosis, and cell senescence via a p53-dependent pathway. Moreover, the inhibitory effect of catalpol on p53 protein expression, DNA damage, apoptosis, and cell senescence was also attenuated by PGC-1*α* or TERT siRNA, indicating that catalpol produces such protective effects via the PGC-1*α*/TERT pathway.

Based on the mitochondria-telomere link, there are positive interactions between PGC-1*α* and TERT, and activating either may benefit to inhibit ROS accumulation, cell apoptosis, and DNA damage [60]. Of interest, after PGC-1*α* knockdown, the effects of catalpol on ROS level were attenuated from 1.52- to 1.37-fold. However, after TERT knockdown, the effects of catalpol on ROS level were only attenuated from 1.51- to 1.44-fold. This can be explained to some extent that the inhibitory effect of catalpol on ROS overgeneration is much more dependent on upregulating PGC-1*α* rather than on upregulating TERT.

## 5. Conclusion

**Figure 9:**
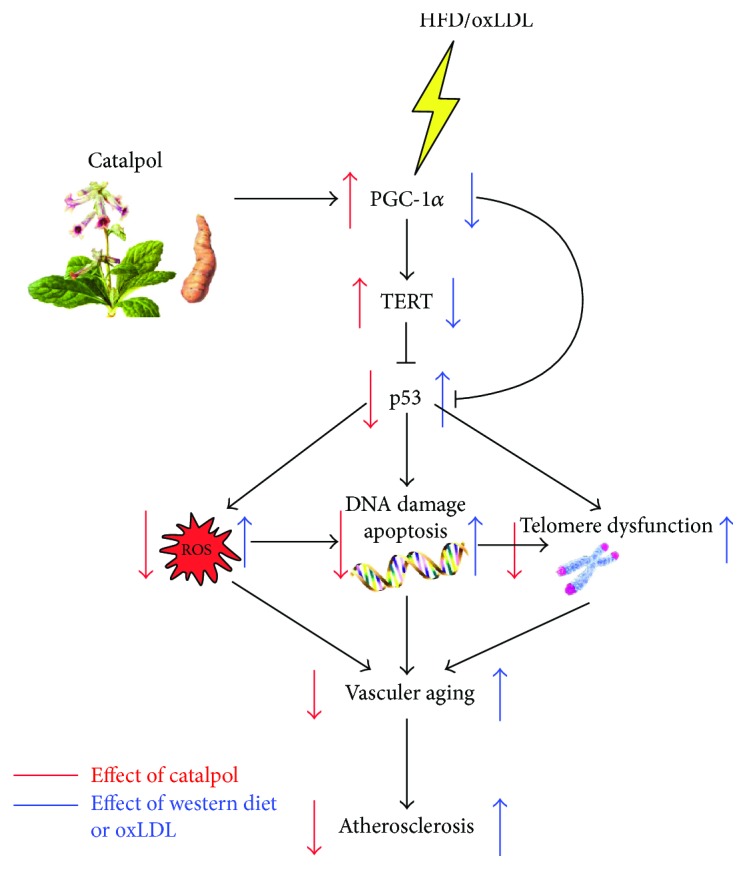
Graphic summary for the mechanism that catalpol ameliorates atherosclerosis through modulating telomere function, DNA damage, and ROS accumulation depending on upregulating the PGC-1*α*/TERT pathway. In HFD-induced atherosclerosis or oxLDL-induced macrophage injury, PGC-1*α* and TERT protein expressions were decreased and p53 protein expression was increased. As a result, ROS overproduction (this ROS may come from NOX2 and NOX4 overexpression and PGC-1*α* inhibition), increased DNA damage, cell apoptosis, and telomere dysfunction occur. Catalpol enhanced PGC-1*α* promoter activity and increased PGC-1*α* expression, thereby suppressing the aforementioned process. These findings indicated a potential mechanism of catalpol on protecting from atherosclerosis. HFD: high-fat diet; oxLDL: oxidized low-density lipoprotein.

Our study indicated the protective effects of catalpol against AS in vitro and in vivo. In HFD-fed LDLr^−/−^ mice, catalpol increased the activities of antioxidant enzymes, improved the lipid profiles in the serum, and reduced the AS lesion area. Moreover, the effects of catalpol were correlated with increased PGC-1*α* and TERT expressions, as well as decreased p53 expression. In an in vitro study, catalpol could attenuate oxLDL-induced oxidative stress and macrophage senescence, including improving telomere function, decreasing DNA damage, and inhibiting ROS overproduction. Using PGC-1*α* and TERT-specific siRNA, we verified that catalpol produced such protective effects via activating the PGC-1*α*/TERT pathway, which is summarized in Figure 9. The present study revealed that the PGC-1*α*/TERT pathway might be a possible therapeutic target in AS and catalpol could lead to suppression of AS via modulating this pathway.

## Abbreviations

AS: Atherosclerosis
PGC-1*α*: Proliferator-activated receptor-*γ* coactivator-1*α*
TERT: Telomerase reverse transcriptase
ROS: Reactive oxygen species
Bcl-2: B-cell lymphoma-2
BCA: Bicinchoninic acid
PMA: Phorbol 12-myristate 13-acetate
MTT: Methyl thiazolyl tetrazolium
DMSO: Dimethyl sulfoxide
oxLDL: Oxidized low-density lipoprotein
TG: Total triglyceride
TC: Total cholesterol
FFA: Liver tissue free fatty acid
SOD: Serum and cell superoxide dismutase
MDA: Malondialdehyde
LDH: Lactate dehydrogenase
GSH: Glutathione
H_2_DCFDA: 2′,7′-Dichlorodihydrofluorescein diacetate
PBS: Phosphate-buffered saline
HE staining: Hematoxylin-eosin staining
IHC: Immunohistochemical
PVDF: Polyvinylidene difluoride
TUNEL: Terminal deoxynucleotidyl transferase-mediated deoxyuridine triphosphate nick-end labeling
8-OHdG: 8-Hydroxydeoxyguanosine
TRAP: Telomeric repeat amplification protocol
SD: Standard deviation
ANOVA: Analysis of variance
SNK: Student-Newman-Keuls
HFD: High-fat diet.

## Supplementary Materials

Supplementary Materials Figure S1: effects of catalpol or oxLDL on cell viability and catalpol-induced protective effects on oxidative stress in oxLDL-treated macrophages. Figure S2: catalpol-induced effects on cell DNA oxidative damage, apoptosis, and DNA damage in oxLDL-treated macrophages. Figure S3: effects of catalpol on DNA oxidative damage, apoptosis, DNA damage, and telomere activity after TERT-specific siRNA treatment.
