# Supplementary material for: Activating the PGC-1α/TERT Pathway by Catalpol Ameliorates Atherosclerosis via Modulating ROS Production, DNA Damage, and Telomere Function: Implications on Mitochondria and Telomere Link
Source: Oxid Med Cell Longev. 2018 Jun 25;2018:2876350. doi: 10.1155/2018/2876350 (PMC6036816; doi:10.1155/2018/2876350)
Supplement: Supplementary Materials — Figure S1: effects of catalpol or oxLDL on cell viability and catalpol-induced protective effects on oxidative stress in oxLDL-treated macrophages. Figure S2: catalpol-induced effects on cell DNA oxidative damage, apoptosis, and DNA damage in oxLDL-treated macrophages. Figure S3: effects of catalpol on DNA oxidative damage, apoptosis, DNA damage, and telomere activity after TERT-specific siRNA treatment. [file 2876350.f1.pdf]

Supplementary Materials

Data description

This supplementary material contains three supplementary figures and its figure legends as following:

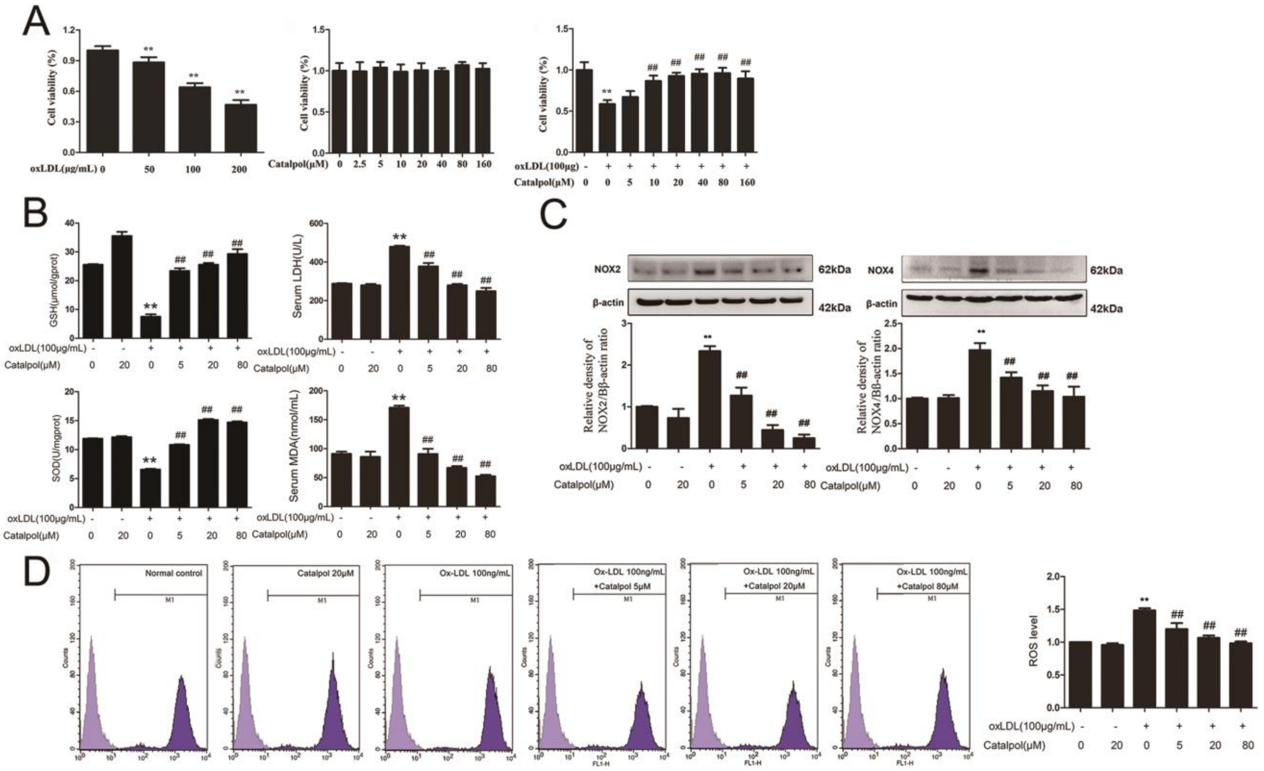

Fig S1.Catalpol reduced oxidative stress in oxLDL-treated macrophages.

THP-1 cells were exposed to PMA (100 ng/mL) for 72 h to induce macrophage formation. Then, macrophages were addressed with oxLDL (0, 100 μg/mL) or catalpol (0, 5, 20, 80 μM) for 24 h. (A) Effect of catalpol and oxLDL on cell viability, n=10. (B) Effect of Catalpol on GSH, SOD, MDA, LDH in oxLDL-treated macrophages, n=10. (C) Effect of catalpol on NOX2, NOX4 protein expression in oxLDL-treated macrophages, n=10. (D) Effect of catalpol on ROS production in oxLDL-treated macrophages, n=10. $^{**}p<0.05$ compared to control group; $^{\#\#}p<0.05$ compared to oxLDL group. Error bars depict the standard deviation.

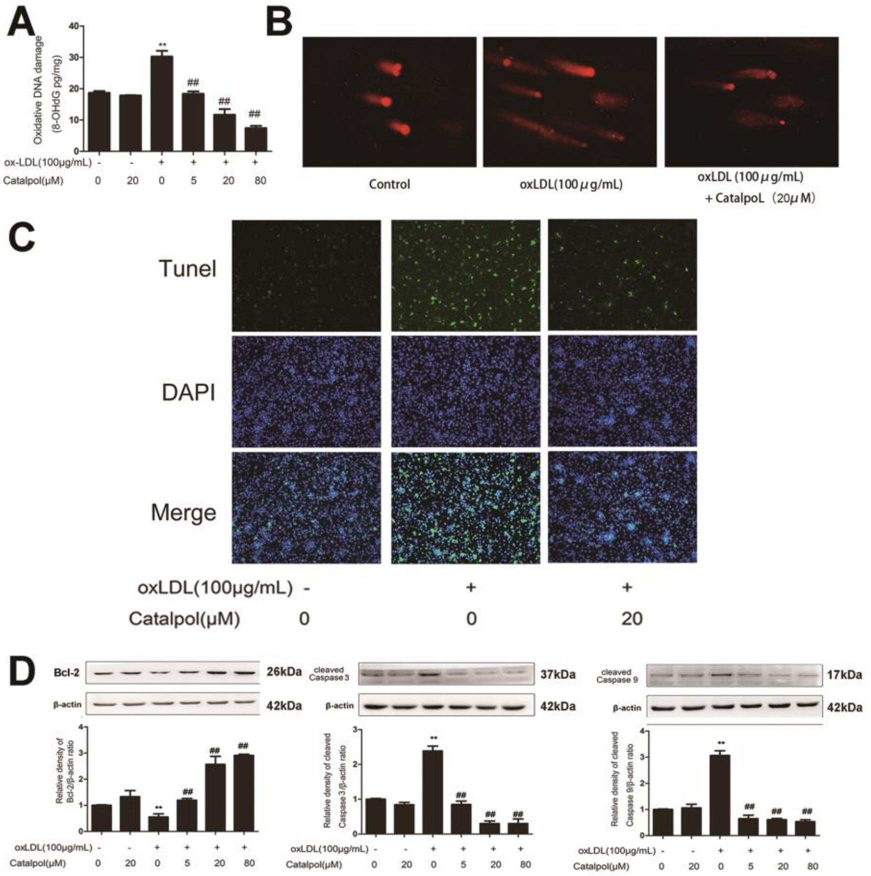

Fig. S2 Catalpol inhibited cell DNA oxidative damage, apoptosis, DNA damage in oxLDL-treated macrophages.

THP-1 cells were exposed to PMA (100 ng/mL) for 72 h to induce macrophage formation. Then, macrophages were treated with oxLDL (0, 100 μg/mL) or catalpol (0, 5, 20, 80 μM) for 24 h. (A) Catalpol inhibited oxidative DNA damage in oxLDL-treated macrophages, n=10. (B) Catalpol inhibited DNA damage in oxLDL-treated macrophages, n=10. (C) Catalpol inhibited cell apoptosis in oxLDL-treated macrophages, n=10. (D) Catalpol inhibited cleaved caspase 3, caspase 9 expression and increased bcl-2 protein expression in oxLDL-treated macrophages, n=10. $^{**}p<0.05$ compared to control group; $^{\#\#}p<0.05$ compared to oxLDL group. Error bars depict the standard deviation.

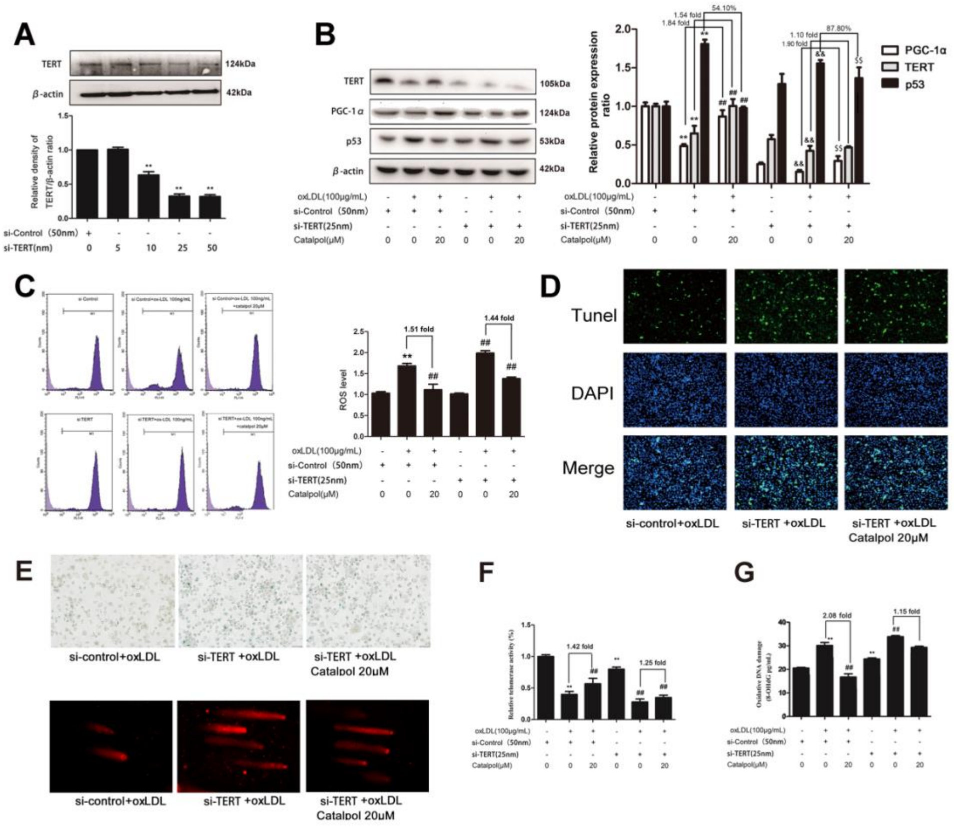

Fig. S3 Catalpol decreases ROS accumulation, DNA damage and ameliorate telomere function through up-regulating TERT expression. THP-1 cells were exposed to PMA (100 ng/mL) for 72 h to induce macrophage formation, and then transfected with small non-targeting RNA for si-control or si-TERT for 12 h. Then, treated with oxLDL or catalpol for additional 24 h, and harvested and analyzed by western blot analysis. (A) Knockdown TERT by its specific siRNA, n=10. (B) Effect of catalpol on increasing PGC-1α, TERT protein expression and decreasing p53 protein expression was attenuated with TERT siRNA, n=10. (C) Effect of catalpol on ROS production with TERT siRNA, n=10. (D) Effects of catalpol on cell apoptosis with TERT siRNA, n=10. (E) Effects of catalpol on cell senescence and DNA damage, n=10. (F) Telomerase activity, n=10. (G) Oxidative DNA damage, n=10. $^{**}p<0.05$

compared to control group; $^{\#\#}p<0.05$ compared to oxLDL group; $^{\&\&}$ $p < 0.05$ compared with si-TERT group, $^{\$\$}$ $p < 0.05$ compared with oxLDL-treated si-TERT group. Error bars depict the standard deviation.
